# Ethyl 2-(3-methyl-5-sulfanyl­idene-4,5-dihydro-1*H*-1,2,4-triazol-4-yl)acetate

**DOI:** 10.1107/S1600536812044716

**Published:** 2012-11-03

**Authors:** Zbigniew Karczmarzyk, Monika Pitucha, Waldemar Wysocki, Andrzej Fruziński, Ewa Olender

**Affiliations:** a Department of Chemistry, Siedlce University, ul. 3 Maja 54, 08-110 Siedlce, Poland; b Department of Organic Chemistry, Faculty of Pharmacy with Division of Medical Analytics, Medical University, ul. Chodźki 4A, 20-093 Lublin, Poland; c Department of General and Ecological Chemistry, Technical University, ul. Żeromskiego 115, 90-924 Łódź, Poland

## Abstract

The title compound, C_7_H_11_N_3_O_2_S, exists in the 5-thioxo tautomeric form. The 1,2,4-triazoline ring is essentially planar, with a maximum deviation of 0.010 (2) Å for the substituted N atom. The ethyl acetate substituent is almost planar, with a maximum deviation of 0.061 (4) Å for the methyl­ene C atom of the eth­oxy group. The angle between the mean plane of this substituent and the mean plane of the 1,2,4-triazoline ring is 89.74 (8)°. In the crystal, mol­ecules are linked by a combination of N—H⋯S, C—H⋯N and C—H⋯O hydrogen bonds into chains parallel to [100].

## Related literature

For background information on the title compound, see: Saadeh *et al.* (2010 ▶); Akhtar *et al.* (2008 ▶); Al-Omar *et al.* (2010 ▶). For the biological activity of 1,2,4-triazoline-thio­nes, see: Pitucha *et al.* (2010 ▶). For their synthesis, see: Bany & Dobosz (1972 ▶). For related structures, see: Kruszynski *et al.* (2007 ▶); Siwek *et al.* (2008 ▶). For graph-set motifs, see: Bernstein *et al.* (1995 ▶).
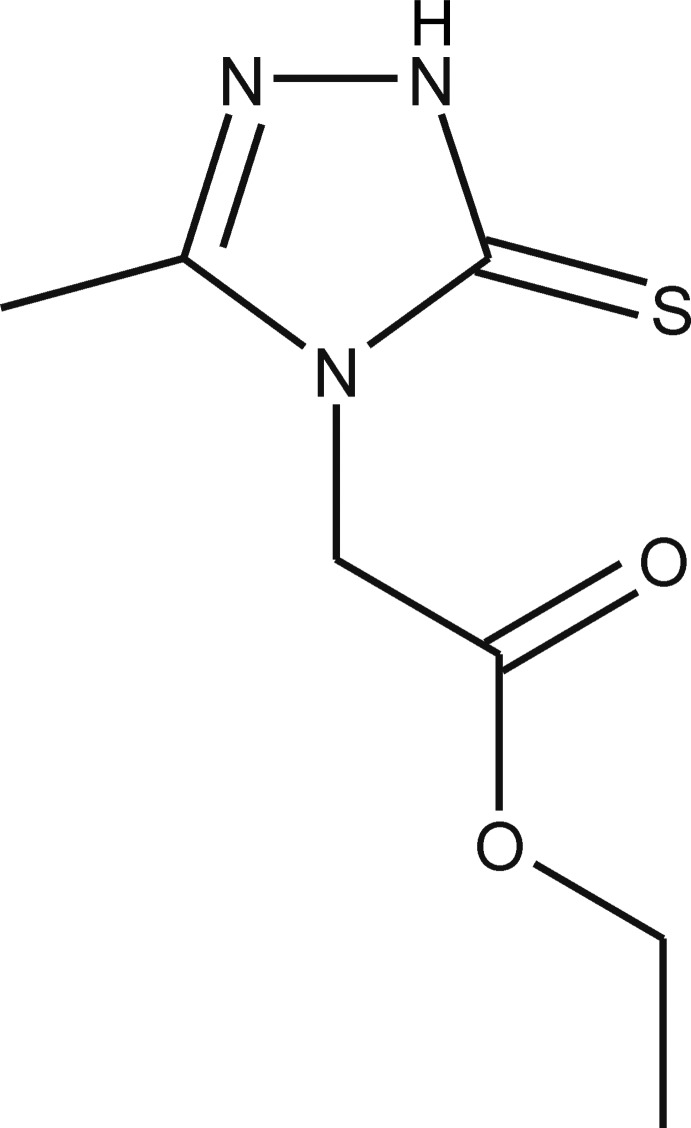


## Experimental

### 

#### Crystal data

C_7_H_11_N_3_O_2_S
*M* *_r_* = 201.25
Monoclinic, 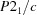

*a* = 6.4438 (19) Å
*b* = 15.2328 (15) Å
*c* = 9.9672 (8) Å
β = 98.416 (19)°
*V* = 967.8 (3) Å^3^
*Z* = 4
Mo *K*α radiation
μ = 0.31 mm^−1^
*T* = 293 K
0.60 × 0.30 × 0.30 mm

#### Data collection

Kuma KM-4 four-circle diffractometer
Absorption correction: ψ scan (North *et al.*, 1968 ▶) *T*_min_ = 0.754, *T*_max_ = 0.869
2979 measured reflections
2837 independent reflections
1571 reflections with *I* > 2σ(*I*)
*R*_int_ = 0.069
2 standard reflections every 100 reflections intensity decay: 8.9%

#### Refinement

*R*[*F* ^2^ > 2σ(*F* ^2^)] = 0.062
*wR*(*F* ^2^) = 0.198
*S* = 0.93
2837 reflections
123 parameters
H atoms treated by a mixture of independent and constrained refinement
Δρ_max_ = 0.64 e Å^−3^
Δρ_min_ = −0.48 e Å^−3^

### 

Data collection: *KM4B8* (Gałdecki *et al.*, 1996 ▶); cell refinement: *KM4B8*; data reduction: *DATAPROC* (Gałdecki *et al.*, 1995 ▶); program(s) used to solve structure: *SHELXS97* (Sheldrick, 2008 ▶); program(s) used to refine structure: *SHELXL97* (Sheldrick, 2008 ▶); molecular graphics: *ORTEP-3 for Windows* (Farrugia, 2012 ▶); software used to prepare material for publication: *SHELXL97* and *WinGX* (Farrugia, 2012 ▶).

## Supplementary Material

Crystal structure: contains datablock(s) I, global. DOI: 10.1107/S1600536812044716/fj2601sup1.cif

Structure factors: contains datablock(s) I. DOI: 10.1107/S1600536812044716/fj2601Isup2.hkl

Supplementary material file. DOI: 10.1107/S1600536812044716/fj2601Isup3.cml

Additional supplementary materials:  crystallographic information; 3D view; checkCIF report

## Figures and Tables

**Table 1:** Hydrogen-bond geometry (Å, °)

*D*—H⋯*A*	*D*—H	H⋯*A*	*D*⋯*A*	*D*—H⋯*A*
N1—H1⋯S6^i^	0.79 (4)	2.56 (4)	3.339 (3)	170 (4)
C8—H8*B*⋯N2^ii^	0.97	2.50	3.407 (3)	155
C13—H13*A*⋯O10^ii^	0.96	2.57	3.482 (5)	159

Symmetry codes: (i) 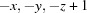; (ii) 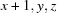.

**Fig. 1.**
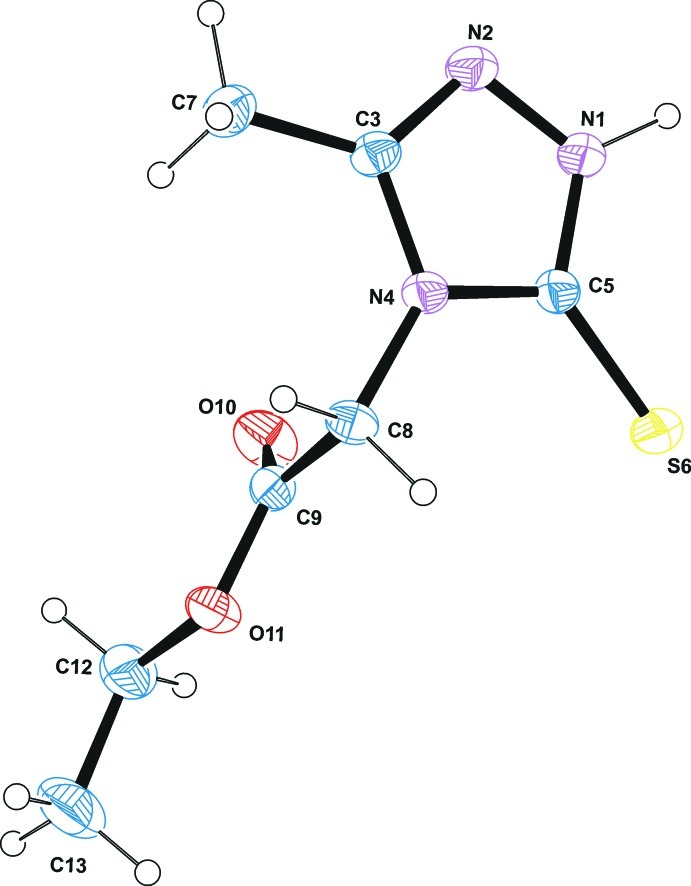
The molecular structure of (I), with atom labels and 30% probability displacement ellipsoids for non-H atoms.

**Fig. 2.**
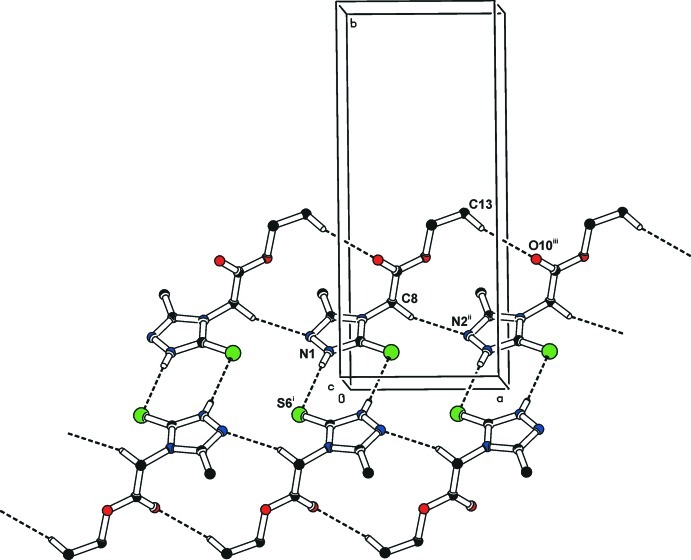
A view of the molecular packing in (I).

## supplementary crystallographic information

### Comment

The 1,2,4-triazoline-thiones were found to have significant antimicrobial action (Saadeh *et al.*, 2010; Akhtar *et al.*, 2008; Al-Omar *et al*., 2010). The title compound, (I), belongs to 3- and 4-substituted derivatives of 1,2,4-triazoline-thiones with potential antituberculosis activity against mycobacterium strains of *Mycobacterium smegmatis*, *Mycobacterium phlei* and *Mycobacterium H37Ra* (Pitucha *et al.*, 2010).

The X-ray analysis of the title compound undertook in order to its structural characterization and to identification of the proper thiol-thione tautomeric form revealed that this compound exists as 5-thioxo tautomer in the crystalline state. The molecular geometry of (I) is very similar to that observed in related structures of 2-(3-methyl-5-thioxo-4,5-dihydro-1*H*-1,2,4-triazol-4-yl)acetic acid (Kruszynski *et al.*, 2007) and 4-[3-(2-methyl-furan-3-yl)-5-thioxo-1,2,4-triazol-4-yl]acetic acid (Siwek *et al.*, 2008). The 1,2,4-triazoline ring is planar to within 0.010 (2) Å. The ethyl acetate chain is almost planar with the most deviating C12 atom from the best C8/C9/O10/O11/C12/C13 plane by 0.061 (4) Å and it adopts a *gauche* conformation in respect to 1,2,4-triazoline ring with the torsion angle C3—N4—C8—C9 of 92.7 (3)°. This conformation is stabilized by the C8—H8B···S6 intramolecular hydrogen bond specified as S(5) in graph set notation (Bernstein *et al.*, 1995).

In the crystal structure, (Fig. 2), the molecules of (I) are linked by a combination of N1—H1···S6, C8—H8B···N2 and C13—H13A···O10 intermolecular hydrogen bond into chains of R^2^_2_(8), R^2^_2_(13) and R^4^_4_(16) edge-fused rings parallel to the [100] direction.

### Experimental

The title compound, (I), was prepared from acetamidrazone hydrochloride and carboethoxymethyl isothiocyanate, according to the method of Bany & Dobosz (1972).

### Refinement

The N-bound H atom was located by difference Fourier synthesis and refined freely. The remaining H atoms were positioned geometrically and treated as riding on their C atoms with C—H distances of 0.93 Å (aromatic), 0.96 Å (CH_2_) and 0.97 Å (CH_3_). All H atoms were assigned *U*_iso_(H) values of 1.5*U*_eq_(N,C)]..

### Crystal data

**Table:**

C_7_H_11_N_3_O_2_S	*F*(000) = 424
*M**_r_* = 201.25	*D*_x_ = 1.381 Mg m^−^^3^
Monoclinic, *P*2_1_/*c*	Melting point = 446–447 K
Hall symbol: -P 2ybc	Mo *K*α radiation, λ = 0.71073 Å
*a* = 6.4438 (19) Å	Cell parameters from 70 reflections
*b* = 15.2328 (15) Å	θ = 2.7–11.9°
*c* = 9.9672 (8) Å	µ = 0.31 mm^−^^1^
β = 98.416 (19)°	*T* = 293 K
*V* = 967.8 (3) Å^3^	Prism, colourless
*Z* = 4	0.60 × 0.30 × 0.30 mm

### Data collection

**Table:**

Kuma KM-4 four-circle diffractometer	1571 reflections with *I* > 2σ(*I*)
Radiation source: fine-focus sealed tube	*R*_int_ = 0.069
Graphite monochromator	θ_max_ = 30.1°, θ_min_ = 2.5°
ω–2θ scans	*h* = −9→8
Absorption correction: ψ scan (North *et al.*, 1968)	*k* = 0→21
*T*_min_ = 0.754, *T*_max_ = 0.869	*l* = 0→14
2979 measured reflections	2 standard reflections every 100 reflections
2837 independent reflections	intensity decay: 8.9%

### Refinement

**Table:**

Refinement on *F*^2^	Primary atom site location: structure-invariant direct methods
Least-squares matrix: full	Secondary atom site location: difference Fourier map
*R*[*F*^2^ > 2σ(*F*^2^)] = 0.062	Hydrogen site location: difference Fourier map
*wR*(*F*^2^) = 0.198	H atoms treated by a mixture of independent and constrained refinement
*S* = 0.93	*w* = 1/[σ^2^(*F*_o_^2^) + (0.1344*P*)^2^] where *P* = (*F*_o_^2^ + 2*F*_c_^2^)/3
2837 reflections	(Δ/σ)_max_ < 0.001
123 parameters	Δρ_max_ = 0.64 e Å^−^^3^
0 restraints	Δρ_min_ = −0.48 e Å^−^^3^

### Special details

**Table:**

Geometry. All esds (except the esd in the dihedral angle between two l.s. planes) are estimated using the full covariance matrix. The cell esds are taken into account individually in the estimation of esds in distances, angles and torsion angles; correlations between esds in cell parameters are only used when they are defined by crystal symmetry. An approximate (isotropic) treatment of cell esds is used for estimating esds involving l.s. planes.
Refinement. Refinement of F^2^ against ALL reflections. The weighted R-factor wR and goodness of fit S are based on F^2^, conventional R-factors R are based on F, with F set to zero for negative F^2^. The threshold expression of F^2^ > 2sigma(F^2^) is used only for calculating R-factors(gt) etc. and is not relevant to the choice of reflections for refinement. R-factors based on F^2^ are statistically about twice as large as those based on F, and R- factors based on ALL data will be even larger.

### Fractional atomic coordinates and isotropic or equivalent isotropic displacement parameters (Å^2^)

**Table:**

	*x*	*y*	*z*	*U*_iso_*/*U*_eq_	
S6	0.29109 (11)	0.08274 (5)	0.48884 (8)	0.0493 (3)	
O10	0.2086 (3)	0.33096 (15)	0.4137 (2)	0.0553 (6)	
O11	0.5003 (3)	0.34124 (13)	0.31670 (19)	0.0423 (5)	
N1	−0.1100 (3)	0.08964 (16)	0.3569 (2)	0.0384 (5)	
H1	−0.152 (6)	0.053 (3)	0.402 (4)	0.058*	
N2	−0.2330 (3)	0.13342 (15)	0.2554 (2)	0.0400 (5)	
N4	0.0940 (3)	0.17783 (14)	0.2764 (2)	0.0324 (4)	
C3	−0.1054 (4)	0.18780 (18)	0.2086 (2)	0.0348 (5)	
C5	0.0896 (4)	0.11528 (17)	0.3742 (2)	0.0338 (5)	
C7	−0.1642 (4)	0.2523 (2)	0.0993 (3)	0.0449 (6)	
H7A	−0.1073	0.2345	0.0198	0.067*	
H7B	−0.1093	0.3089	0.1282	0.067*	
H7C	−0.3143	0.2555	0.0788	0.067*	
C8	0.2820 (4)	0.22340 (18)	0.2511 (2)	0.0348 (5)	
H8A	0.2677	0.2399	0.1562	0.052*	
H8B	0.4014	0.1842	0.2700	0.052*	
C9	0.3210 (4)	0.30402 (17)	0.3374 (2)	0.0347 (5)	
C12	0.5476 (5)	0.4245 (2)	0.3862 (4)	0.0553 (8)	
H12A	0.5543	0.4166	0.4833	0.083*	
H12B	0.4386	0.4670	0.3561	0.083*	
C13	0.7523 (7)	0.4562 (3)	0.3544 (5)	0.0743 (11)	
H13A	0.8607	0.4158	0.3902	0.111*	
H13B	0.7818	0.5130	0.3944	0.111*	
H13C	0.7469	0.4604	0.2578	0.111*	

### Atomic displacement parameters (Å^2^)

**Table:**

	*U*^11^	*U*^22^	*U*^33^	*U*^12^	*U*^13^	*U*^23^
S6	0.0354 (4)	0.0521 (4)	0.0567 (5)	−0.0037 (3)	−0.0051 (3)	0.0156 (3)
O10	0.0513 (12)	0.0536 (13)	0.0663 (13)	−0.0048 (10)	0.0263 (10)	−0.0162 (10)
O11	0.0342 (9)	0.0406 (10)	0.0530 (11)	−0.0086 (8)	0.0094 (7)	−0.0052 (8)
N1	0.0306 (10)	0.0387 (12)	0.0460 (12)	−0.0034 (9)	0.0064 (8)	0.0086 (10)
N2	0.0278 (10)	0.0463 (12)	0.0457 (12)	−0.0014 (9)	0.0046 (8)	0.0046 (10)
N4	0.0247 (9)	0.0336 (10)	0.0392 (10)	−0.0001 (8)	0.0061 (7)	0.0002 (8)
C3	0.0280 (11)	0.0398 (13)	0.0367 (12)	0.0024 (10)	0.0052 (9)	0.0003 (10)
C5	0.0300 (11)	0.0302 (11)	0.0411 (13)	−0.0001 (9)	0.0051 (9)	0.0001 (10)
C7	0.0352 (14)	0.0523 (16)	0.0467 (15)	0.0049 (12)	0.0045 (11)	0.0091 (12)
C8	0.0268 (11)	0.0411 (13)	0.0384 (12)	−0.0036 (10)	0.0115 (9)	−0.0008 (10)
C9	0.0329 (12)	0.0355 (12)	0.0360 (12)	0.0001 (10)	0.0062 (9)	0.0038 (10)
C12	0.0517 (17)	0.0402 (15)	0.072 (2)	−0.0077 (13)	0.0029 (15)	−0.0088 (15)
C13	0.063 (2)	0.057 (2)	0.105 (3)	−0.0248 (18)	0.019 (2)	−0.007 (2)

### Geometric parameters (Å, º)

**Table:**

S6—C5	1.674 (3)	C7—H7A	0.9600
O10—C9	1.197 (3)	C7—H7B	0.9600
O11—C9	1.330 (3)	C7—H7C	0.9600
O11—C12	1.455 (4)	C8—C9	1.499 (4)
N1—C5	1.331 (3)	C8—H8A	0.9700
N1—N2	1.364 (3)	C8—H8B	0.9700
N1—H1	0.79 (4)	C12—C13	1.482 (5)
N2—C3	1.301 (3)	C12—H12A	0.9700
N4—C5	1.367 (3)	C12—H12B	0.9700
N4—C3	1.369 (3)	C13—H13A	0.9600
N4—C8	1.450 (3)	C13—H13B	0.9600
C3—C7	1.475 (4)	C13—H13C	0.9600
			
C9—O11—C12	115.0 (2)	N4—C8—H8A	109.3
C5—N1—N2	113.5 (2)	C9—C8—H8A	109.3
C5—N1—H1	123 (3)	N4—C8—H8B	109.3
N2—N1—H1	124 (3)	C9—C8—H8B	109.3
C3—N2—N1	104.3 (2)	H8A—C8—H8B	108.0
C5—N4—C3	108.3 (2)	O10—C9—O11	124.9 (3)
C5—N4—C8	124.3 (2)	O10—C9—C8	125.4 (2)
C3—N4—C8	127.5 (2)	O11—C9—C8	109.7 (2)
N2—C3—N4	110.5 (2)	O11—C12—C13	108.2 (3)
N2—C3—C7	125.5 (2)	O11—C12—H12A	110.1
N4—C3—C7	124.0 (2)	C13—C12—H12A	110.1
N1—C5—N4	103.4 (2)	O11—C12—H12B	110.1
N1—C5—S6	129.8 (2)	C13—C12—H12B	110.1
N4—C5—S6	126.74 (19)	H12A—C12—H12B	108.4
C3—C7—H7A	109.5	C12—C13—H13A	109.5
C3—C7—H7B	109.5	C12—C13—H13B	109.5
H7A—C7—H7B	109.5	H13A—C13—H13B	109.5
C3—C7—H7C	109.5	C12—C13—H13C	109.5
H7A—C7—H7C	109.5	H13A—C13—H13C	109.5
H7B—C7—H7C	109.5	H13B—C13—H13C	109.5
N4—C8—C9	111.52 (19)		
			
C5—N1—N2—C3	0.0 (3)	C8—N4—C5—N1	−177.5 (2)
N1—N2—C3—N4	1.2 (3)	C3—N4—C5—S6	−176.9 (2)
N1—N2—C3—C7	−178.1 (3)	C8—N4—C5—S6	3.8 (4)
C5—N4—C3—N2	−1.9 (3)	C5—N4—C8—C9	−88.1 (3)
C8—N4—C3—N2	177.3 (2)	C3—N4—C8—C9	92.7 (3)
C5—N4—C3—C7	177.4 (2)	C12—O11—C9—O10	−5.1 (4)
C8—N4—C3—C7	−3.3 (4)	C12—O11—C9—C8	175.1 (2)
N2—N1—C5—N4	−1.1 (3)	N4—C8—C9—O10	−3.2 (4)
N2—N1—C5—S6	177.5 (2)	N4—C8—C9—O11	176.7 (2)
C3—N4—C5—N1	1.8 (3)	C9—O11—C12—C13	179.2 (3)

### Hydrogen-bond geometry (Å, º)

**Table:**

*D*—H···*A*	*D*—H	H···*A*	*D*···*A*	*D*—H···*A*
N1—H1···S6^i^	0.79 (4)	2.56 (4)	3.339 (3)	170 (4)
C8—H8*B*···N2^ii^	0.97	2.50	3.407 (3)	155
C13—H13*A*···O10^ii^	0.96	2.57	3.482 (5)	159

Symmetry codes: (i) −*x*, −*y*, −*z*+1; (ii) *x*+1, *y*, *z*.
